# No Effect of Ego Depletion on Risk Taking

**DOI:** 10.1038/s41598-019-46103-0

**Published:** 2019-07-05

**Authors:** Lina Koppel, David Andersson, Daniel Västfjäll, Gustav Tinghög

**Affiliations:** 1 JEDI Lab, Division of Economics, Department of Management and Engineering, Linköping University, Linköping, Sweden; 2 Center for Social and Affective Neuroscience, Department of Clinical and Experimental Medicine, Linköping University, Linköping, Sweden; 3 Division of Psychology, Department of Behavioral Sciences and Learning, Linköping University, Linköping, Sweden; 4 Decision Research, Eugene, OR USA; 5 The National Center for Priority Setting in Health Care, Department of Medical and Health Sciences, Linköping University, Linköping, Sweden

**Keywords:** Reward, Human behaviour

## Abstract

We investigated the effect of ego depletion on risk taking. Specifically, we conducted three studies (total *n* = 1,716) to test the prediction that ego depletion results in decisions that are more strongly in line with prospect theory, i.e., that ego depletion reduces risk taking for gains, increases risk taking for losses, and increases loss aversion. Ego depletion was induced using two of the most common manipulations from previous literature: the letter ‘e’ task (Studies 1 and 3) and the Stroop task (Study 2). Risk taking was measured using a series of standard, incentivized economic decision-making tasks assessing risk preferences in the gain domain, risk preferences in the loss domain, and loss aversion. None of the studies revealed a significant effect of ego depletion on risk taking. Our findings cast further doubts about the ability of ego-depletion manipulations to affect actual behavior in experimental settings.

## Introduction

Self-control is a key factor for success in many areas of life, including financial behavior^1^ and academic success^2^. In this paper, we explore the effect of temporary depletion of one’s self-control resources—the state known as ego depletion—on risk taking, a core component of everyday behavior and decision making. It is a common belief that inhibition of controlled (i.e., System 2) processing influences risk taking, yet results from previous studies are mixed and ego depletion as a phenomenon has been a topic of debate in recent years. We here report three studies investigating the prediction that ego depletion results in decisions that are more strongly in line with prospect theory, i.e., that ego depletion reduces risk taking for gains, increases risk taking for losses, and increases loss aversion. Study 1 reports data collected by our lab as part of the Hagger *et al*. multi-lab registered replication report (RRR) on the ego-depletion effect^3^. Study 2 extends those findings using a larger sample and a manipulation that has been shown to more effectively induce ego depletion. Study 3 further shows the robustness of our results in a high-powered online study.

According to dual-process theories, decisions are the result of an interaction between intuitive (System 1) and deliberative (System 2) processes^4–8^. System 1 is commonly characterized as fast, automatic, and effortless, whereas System 2 is characterized as slow, controlled, and effortful. To study these processes, researchers typically use manipulations that inhibit System 2, thereby increasing reliance on System 1. Common manipulations include time pressure, cognitive load, and cognitive depletion (ego depletion), which is the approach used here.

System 1 gives rise to a number of automatic biases in everyday behavior and decision making. With regards to risk taking, System 1 is arguably reflected in the S-shaped value function of prospect theory^8^. That is, people tend to be risk averse for gains and risk taking for losses (known as the *reflection effect*) and more sensitive to losses relative to gains (known as *loss aversion*)^9^. Increasing reliance on System 1 should enhance these behavioral tendencies and make people less likely to make decisions that maximize expected value. In line with this prediction, previous studies have shown that time pressure (compared to time delay)^10^ and stress (compared to no stress)^11^ reduce risk taking for gains and increase risk taking for losses, indicating that inhibiting System 2 leads to an increased reflection effect of prospect theory. Similar results have been found when comparing participants who score low on the Cognitive Reflection Test (CRT; which indicates that they rely more on System 1) to those who score high (who rely more on System 2)^12^. Furthermore, under time pressure but not time delay, participants’ risky choices are predicted by increased skin conductance levels, suggesting that time pressure increases reliance on affective (System 1) signals^13^. Although different types of System 1–System 2 manipulations are assumed to have similar effects on behavior, no previous study has explored whether inhibiting System 2 using ego depletion makes people behave more in line with prospect theory.

Ego depletion refers to the phenomenon that exerting self-control in one task reduces performance in a second task that also requires self-control. For example, participants who have resisted the temptation to eat chocolate give up sooner on a difficult problem-solving task, compared to participants who have not^14^. Ego depletion is arguably a suitable manipulation of System 1–System 2 processing, because System 2 involves control and effort and relies on self-regulatory resources. Depleting or reducing the self-regulatory resources should inhibit System 2 and make decisions more intuitive and System 1 based. To induce ego depletion, we used two of the most common tasks from previous literature: the letter ‘e’ task (Studies 1 and 3) and the Stroop task (Study 2). These tasks have been recommended in recent meta-analyses because of their effectiveness in inducing a state of ego depletion^15–17^. Using an appropriate depletion task was especially important given that ego depletion has been a topic of much debate in recent years. We also include a large sample of participants. Thus, we give ego depletion the best possible chance to produce an effect.

Although no previous study has tested the prediction that ego depletion results in more prospect-theory-like behavior, previous studies have investigated the effect of ego depletion on risk taking more generally. Results from these studies are mixed (see Supplementary Table S1). Most published studies have suggested that ego depletion increases risk taking^18–23^. Specifically, ego depletion has been shown to increase self-reported sensation seeking and risk taking^19^ as well as actual risk taking in both incentivized^20,21^ and unincentivized^18–20,22,23^ tasks. However, some studies have found the opposite effect: ego depletion reduces risk taking^24–26^. Yet other studies have found that the effect of ego depletion on risk taking depends on factors such as trait self-control^27^ (but see two other studies that found no significant interaction between state and trait self-control^20,28^), the amount of effort required in the risk task^29^, and using intuition to guide decision making^30^. Given these inconsistencies, it is perhaps unsurprising that a recent high-powered (*n* = 308) study failed to find any effect of ego depletion on risk taking in an incentivized multiple choice list task^28^.

In sum, previous studies on the effect of ego depletion on risk taking have used a variety of manipulations of ego depletion and a variety of measures of risk taking, and sample sizes have generally been small (with one exception^28^). Therefore, the literature might suffer from some of the same potential issues as the ego depletion literature at large, including small-study bias^31–33^. Furthermore, with few exceptions^21,28^, previous studies have not used risk-taking measures that are specifically designed to inform theoretical models of economic decision making. Most importantly, no study has distinguished between gains and losses, which is critical in prospect theory. Therefore, the existing literature cannot address the question of whether ego depletion enhances the automatic biases described by the value function of prospect theory—i.e., whether ego depletion enhances the reflection effect and increases loss aversion. We here specifically test this prediction.

Across three preregistered studies, we investigate the effect of ego depletion on risk taking in a series of standard, incentivized economic decision-making tasks that assess risk preferences in the gain domain, risk preferences in the loss domain, and loss aversion. Study 1 reports data collected by our lab as part of the Hagger *et al*. RRR^3^. Participants performed a computerized version of the letter ‘e’ task, followed by a set of four manipulation check questions, a multi-source interference task (MSIT; which was used as the outcome measure in Hagger *et al*.), a second set of manipulation check questions, and the risk tasks. Results from the letter ‘e’ task, the first set of manipulation checks, and the MSIT were reported in Hagger *et al*.^3^; the second set of manipulation checks and the risk-taking tasks were implemented by our lab only and therefore have not previously been reported. However, all parts of the study were included in our lab’s preregistered protocol.

To be sure of the results from Study 1, we conducted two additional studies in which we included a larger sample (Study 2: *n* = 230; Study 3: *n* = 1,389) and fine-tuned the risk-taking tasks to increase number of trials in the range where there seemed to be a tendency toward an effect in Study 1. In addition, we addressed some concerns that were raised regarding the Hagger *et al*. RRR. The main critique of the RRR was that the study failed to properly manipulate ego depletion. Specifically, the computerized version of the letter ‘e’ task omitted the habit-forming first phase, which may have reduced its effectiveness in reducing performance on the second task^34,35^. Re-analyses of the Hagger *et al*. results suggest that ego depletion can be induced given appropriate depletion tasks^36^. In Study 2, we induced ego depletion using a manipulation that has been recommended in recent literature^16,17^ and that had a significant effect in a recent multi-lab replication project^37^—the Stroop task. In Study 3, we induced ego depletion using a version of the letter ‘e’ task that included the habit-forming first phase and that was based on materials from a recent multi-lab replication project which was conducted in response to the Hagger *et al*. RRR^34,38^.

## Results

### Study 1

#### Manipulation check

Following the letter ‘e’ task, participants in the depletion condition reported significantly greater effort (*t*_95_ = 3.63, *p* < 0.001), difficulty (*t*_95_ = 11.28, *p* < 0.0001), and frustration (*t*_95_ = 3.67, *p* < 0.001), but not fatigue (*t*_95_ = 1.26, *p* = 0.209), than participants in the control condition (for means, see Table S2 in the Supplementary Information). However, after the MSIT, there was no difference in the level of effort, fatigue, or frustration between the two conditions (all *p*s > 0.250) and a difference in the opposite direction in difficulty (*t*_95_ = –2.48, *p* = 0.015).

#### Risk taking

**Figure 1:**
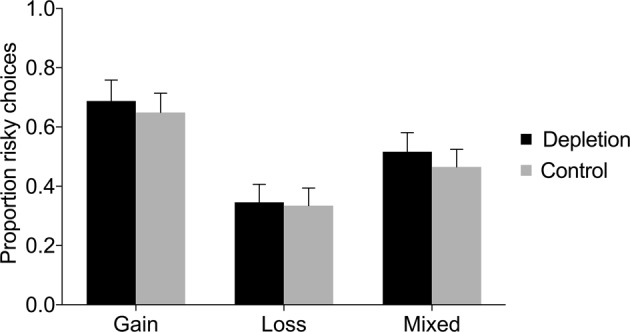
Proportion of risky choices in the gain domain, loss domain, and mixed gambles, as a function of condition (depletion vs. control) in Study 1. Error bars represent 95% CIs.

**Figure 2:**
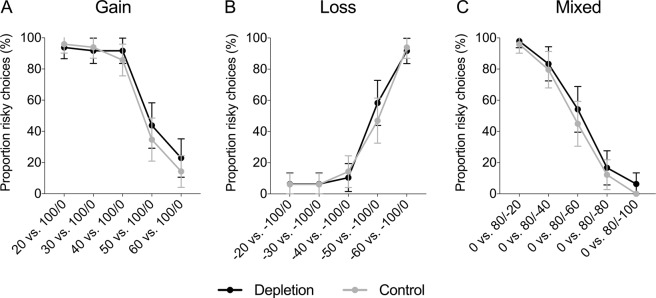
Proportion (%) of risky choices per trial in the (**A**) gain domain, (**B**) loss domain, and (**C**) mixed gambles, as a function of condition (depletion vs. control) in Study 1. Error bars represent 95% CIs.

Figure 1 displays the proportion of risky choices in each condition (depletion and control) in the gain domain, loss domain, and mixed gambles. Independent samples t-tests and Mann-Whitney U tests revealed no significant difference in the proportion of risky choices between the depletion and control condition, neither in the gain domain, nor in the loss domain, nor in the mixed gambles (see Table 1). Regression analyses controlling for age and gender found no significant effects either (see Table 2). In the gain domain, there was an unexpected effect of gender suggesting that women were more risk taking than men (*β* = 0.10, *SE* = 0.05, *p* = 0.033); however, there was no interaction between gender and condition (see Supplementary Table S3). Chi-square tests investigating each trial separately also revealed no significant difference in risk taking between ego depletion and control (see Fig. 2 and Supplementary Table S4).

**Table 1:** Significance tests of the difference in the proportion of risky choices in the depletion vs. control condition in the gain domain, loss domain, and mixed gambles in Study 1.

	Depletion		Control		Independent-samples t-test			Mann-Whitney U
	*M*	[95% CI]	*M*	[95% CI]	*t*	*p*	*d*	*p*
Gain	0.69	[0.62, 0.76]	0.65	[0.58, 0.71]	0.81	0.421	0.16	0.270
Loss	0.35	[0.29, 0.41]	0.33	[0.28, 0.39]	0.26	0.792	0.05	0.754
Mixed	0.52	[0.45, 0.58]	0.47	[0.41, 0.52]	1.18	0.240	0.24	0.291

**Table 2:** Regression analyses of risky choices in Study 1.

	Gain	Loss	Mixed
Depletion	0.042 (0.046)	0.014 (0.041)	0.049 (0.042)
Female	0.104* (0.048)	0.079 (0.049)	−0.059 (0.046)
Age	0.005 (0.010)	0.004 (0.009)	−0.000 (0.009)
Constant	0.495* (0.224)	0.225 (0.210)	0.493* (0.207)

*Notes*. This table reports OLS coefficient estimates (robust standard errors in parentheses). The dependent variable is the proportion of risky choices. “Depletion” is a dummy for the depletion condition. “Female” is a gender dummy. “Age” is the participant’s age in years.

*p < 0.05, **p < 0.01, ***p < 0.001.

### Study 2

#### Manipulation check

Following the Stroop task, participants in the depletion condition reported significantly greater effort (*t*_228_ = 4.24, *p* < 0.0001), difficulty (*t*_228_ = 7.45, *p* < 0.0001), and fatigue (*t*_228_ = 2.02, *p* = 0.045), but not frustration (*t*_228_ = −0.56, *p* = 0.579), than participants in the control condition (for means, see Supplementary Table S5).

#### Risk taking

**Figure 3:**
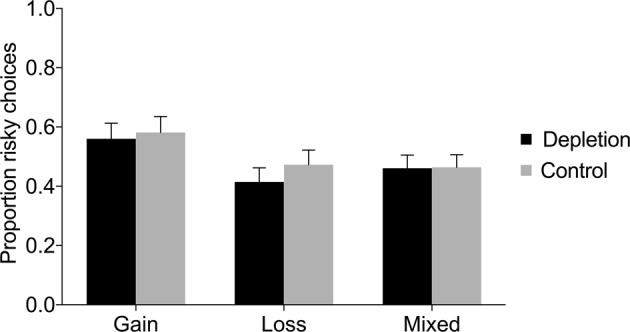
Proportion of risky choices in the gain domain, loss domain, and mixed gambles, as a function of condition (depletion vs. control) in Study 2. Error bars represent 95% CIs.

**Figure 4:**
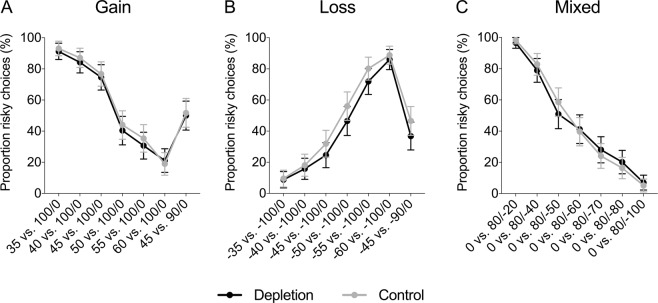
Proportion (%) of risky choices per trial in the (**A**) gain domain, (**B**) loss domain, and (**C**) mixed gambles, as a function of condition (depletion vs. control) in Study 2. Error bars represent 95% CIs.

Figure 3 displays the proportion of risky choices in each condition (depletion vs. control) in the gain domain, loss domain, and mixed gambles. There was no significant difference in the proportion of risky choices between the depletion and control condition, neither in the gain domain, nor in the loss domain, nor in the mixed gambles (see Table 3). Regression analyses controlling for age and gender found no significant effects either (see Table 4); however, note that the regression analyses were performed on a smaller sample, since not all participants reported age and gender. In the mixed gambles, there was a significant effect of gender suggesting that women were less risk taking than men (*β* = −0.09, *SE* = 0.03, *p* = 0.007); however, there was no interaction between gender and condition (see Supplementary Table S6). Chi-square tests investigating each trial separately also revealed no significant difference in risk taking between depletion and control (see Fig. 4 and Supplementary Table S7).

**Table 3:** Significance tests of the difference in the proportion of risky choices in the depletion vs. control condition in the gain domain, loss domain, and mixed gambles in Study 2.

	Depletion		Control		Independent-samples t-test			Mann-Whitney U
	*M*	[95% CI]	*M*	[95% CI]	*t*	*p*	*d*	*p*
Gain	0.56	[0.51, 0.61]	0.58	[0.53, 0.64]	−0.55	0.581	0.07	0.511
Loss	0.41	[0.37, 0.46]	0.47	[0.42, 0.52]	−1.68	0.094	0.22	0.061
Mixed	0.46	[0.42, 0.51]	0.46	[0.42, 0.51]	−0.10	0.920	0.01	0.817

**Table 4:** Regression analyses of risky choices in Study 2.

	Gain	Loss	Mixed
Depletion	−0.010 (0.043)	−0.049 (0.039)	−0.003 (0.035)
Female	0.060 (0.043)	−0.029 (0.038)	−0.093** (0.034)
Age	0.000 (0.004)	0.002 (0.039)	−0.001 (0.003)
Constant	0.521*** (0.104)	0.441*** (0.097)	0.532*** (0.073)

*Notes*. This table reports OLS coefficient estimates (robust standard errors in parentheses). The dependent variable is the proportion of risky choices. “Depletion” is a dummy for the depletion condition. “Female” is a gender dummy. “Age” is the participant’s age in years.

*p < 0.05, **p < 0.01, ***p < 0.001.

#### Pooled data from studies 1 and 2

In an additional analysis that was not specified in the preregistration, the data from studies 1 and 2 were pooled in order to investigate an overall effect of ego depletion on risk taking. This analysis revealed no significant effect (see Supplementary Table S8).

### Study 3

#### Manipulation check

Following the letter ‘e’ task, participants in the depletion condition reported significantly greater difficulty (*t*_1387_ = 8.11, *p* < 0.001), fatigue (*t*_1387_ = 2.09, *p* = 0.037), and frustration (*t*_1387_ = 3.27, *p* = 0.001), but not effort (*t*_1387_ = 1.24, *p* = 0.215), than participants in the control condition (for means, see Supplementary Table S9).

#### Risk taking

**Figure 5:**
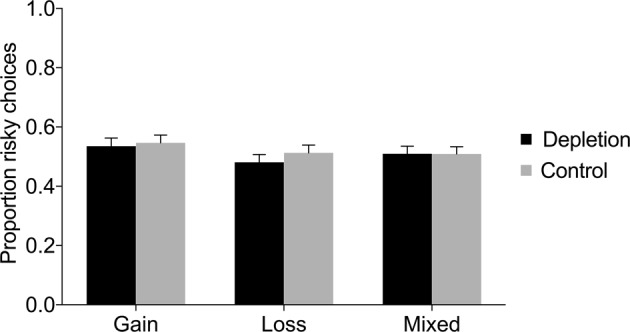
Proportion of risky choices in the gain domain, loss domain, and mixed gambles, as a function of condition (depletion vs. control) in Study 3. Error bars represent 95% CIs.

**Figure 6:**
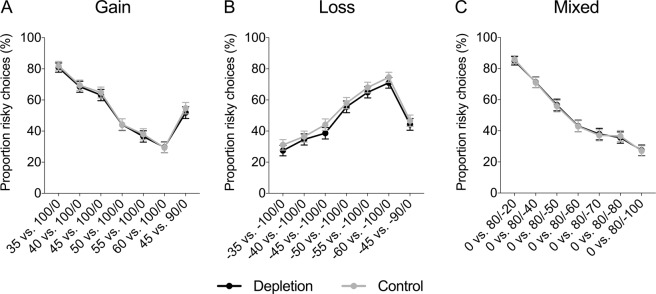
Proportion (%) of risky choices per trial in the (**A**) gain domain, (**B**) loss domain, and (**C**) mixed gambles, as a function of condition (depletion vs. control) in Study 3. Error bars represent 95% CIs.

Figure 5 displays the proportion of risky choices in each condition (depletion vs. control) in the gain domain, loss domain, and mixed gambles. There was no significant difference in the proportion of risky choices between the depletion and control condition, neither in the gain domain, nor in the loss domain, nor in the mixed gambles (see Table 5). Regression analyses controlling for age and gender found no significant effects either (see Table 6). In the gain domain, there was a significant effect of gender suggesting that women were less risk taking than men (*β* = −0.04, *SE* = 0.02, *p* = 0.040); however, there was no interaction between gender and condition (see Supplementary Table S10). Chi-square tests investigating each trial separately also revealed no significant difference in risk taking between depletion and control, except on one trial (see Fig. 6 and Supplementary Table S11).

**Table 5:** Significance tests of the difference in the proportion of risky choices in the depletion vs. control condition in the gain domain, loss domain, and mixed gambles in Study 3.

	Depletion		Control		Independent-samples t-test			Mann-Whitney U
	*M*	[95% CI]	*M*	[95% CI]	*t*	*p*	*d*	*p*
Gain	0.54	[0.34, 0.38]	0.55	[0.34, 0.38]	−0.59	0.558	0.03	0.587
Loss	0.48	[0.33, 0.37]	0.51	[0.34, 0.38]	−1.68	0.093	0.09	0.090
Mixed	0.51	[0.32, 0.35]	0.51	[0.32, 0.35]	0.05	0.960	0.003	0.948

**Table 6:** Regression analyses of risky choices in Study 3.

	Gain	Loss	Mixed
Depletion	−0.016 (0.019)	−0.027 (0.019)	0.005 (0.018)
Female	−0.040* (0.020)	0.034 (0.019)	0.017 (0.018)
Age	−0.001 (0.001)	0.001 (0.001)	−0.001 (0.001)
Constant	0.609*** (0.035)	0.441*** (0.036)	0.532*** (0.033)

*Notes*. This table reports OLS coefficient estimates (robust standard errors in parentheses). The dependent variable is the proportion of risky choices. “Depletion” is a dummy for the depletion condition. “Female” is a gender dummy. “Age” is the participant’s age in years.

*p < 0.05, **p < 0.01, ***p < 0.001

## Discussion

We investigated the effect of ego depletion on risk taking. Across three preregistered studies (total *n* = 1,716), we find no evidence that ego depletion results in more prospect-theory-like behavior. Specifically, participants who had performed a difficult version of the letter ‘e’ task (Studies 1 and 3) or the Stroop task (Study 2) were not significantly less risk taking in the gain domain, more risk taking in the loss domain, or more loss averse, compared to participants who had performed an easy version.

One possible explanation for our null findings is that the effect of ego depletion itself is either non-existent or too small to produce a consistent effect on risk taking, at least in the lab. This suggestion might seem counterintuitive given that the manipulation checks indicated that participants in the depletion condition exerted more effort (Study 1 and 2), reported that the task was more difficult (Study 1–3) and felt more frustrated (Study 1 and 3) and fatigued (Study 2 and 3) than control participants. However, self-reported depletion is not necessarily a measure of actual depletion of one’s self-control resources. Ego depletion is commonly linked to the strength model of self-control, which posits that exerting self-control in one task reduces performance on a second task that also requires self-control^14^. The theory has received extensive empirical support and an initial meta-analysis of 198 independent tests of ego depletion revealed a medium-to-large effect size^15^. However, results from recent multi-lab replications have been mixed. Some have found effects consistent with a null effect^3,39^; others have found small but significant effects in the predicted direction^37,38^. Part of the discussion has centered around the specific tasks that are used to induce and measure ego depletion, and efforts have been made to establish which tasks are most effective^16,17^. However, despite using manipulations based on recent meta-analyses and recommendations, our studies failed to find a significant effect on risk taking. Given the current state of the ego depletion literature, we cannot rule out the possibility that the ego depletion effect itself is non-existent or very small or that it depends on factors not measured in the present studies.

An alternative explanation for our null findings is that participants were indeed depleted to some extent but that they were able to continue to exert self-control in the risk task because the possibility of a monetary reward motivated them to do so. Previous research suggests that increasing participants’ motivation to perform a task that requires self-control can improve their performance. For example, depleted participants who are paid well to drink a bad-tasting beverage (which arguably requires self-control) drink as much of it as non-depleted participants, but depleted participants who are paid poorly drink less of the bad-tasting beverage than non-depleted participants (as expected based on the ego-depletion literature)^40^. Reminding participants of the importance of the task can also ameliorate ego depletion^41^. In light of these and similar findings, one might argue that the reason we found no effect is that the outcome measure, the risk task, was incentivized. However, studies on ego depletion and risk taking have used both incentivized and non-incentivized tasks and some have found effects in one direction, whereas others have found effects in the other direction—and yet others have found no effect at all^18–30^. Therefore, it seems unlikely that the incentivized nature of the risk task is the sole reason for our null results.

A third possibility is that ego depletion has no effect on risk taking because decision making under risk is not dependent on self-control. This suggestion is arguably at odds with dual-process models of decision making, which place a central role of self-control in System 2 processing. Compared to the automatic, intuitive System 1, System 2 is controlled and effortful and relies on self-regulatory resources. When self-control is depleted, System 2 can no longer override System 1, which means that automatic biases such as the reflection effect of prospect theory are enhanced. Indeed, participants who make decisions under time pressure, compared to time delay, are more risk seeking for losses and less risk seeking for gains^10^ and rely more on affective (System 1) signals^13^. Similar results have been found for stress (which arguably also inhibits System 2)^11^ and when comparing participants who habitually rely more on System 1 and System 2 processing, respectively^12^. The present research failed to find such effects and thereby failed to provide support for the prediction that ego depletion results in decisions that are more strongly in line with prospect theory. Although different manipulations of System1–System 2 processing are assumed to have similar effects on behavior, it is possible that different manipulations seeking to inhibit System 2 have different behavioral effects. For example, acute pain (which arguably also inhibits System 2) has been shown to increase risk taking overall (especially for gains), which is somewhat at odds with the prediction that inhibition of System 2 processes results in more prospect-theory-like behavior^42^.

We suggest that future studies investigate self-control depletion that may arise after more prolonged exertion of effort (also known as mental fatigue^43^) or after a long series of previous decisions (decision fatigue^44^). For example, judges are less likely to grant parole to prisoners^45^ and surgeons are less likely to decide to operate^46^ toward the end of their work shift. Such approaches could inform both the self-control literature and theories of decision making.

## Methods

### Study 1

The preregistered protocol and data are available at the Open Science Framework (OSF)^47^.

#### Participants

102 participants were recruited from a subject pool at Linköping University for an experiment on “word and number recognition and reaction time”. They signed up using ORSEE^48^. Three participants were excluded because they did not fulfil the language requirement; an additional two were excluded due to technical issues administering the risk-taking task. The final sample consisted of usable data from 97 participants (33% female; age 23.15 ± 2.55 years [m ± SD]). All participants gave their written informed consent and were compensated with 100 SEK (approx. 12 USD) in addition to the payoff from one randomly selected decision. All methods were carried out in accordance with relevant ethical guidelines and regulations. According to guidelines from the Swedish research council concerning the Ethical Review of Research Involving Humans (SFS 2003:460), approval from an ethics committee is not required for behavioral research such as this study.

#### Materials and procedure

Participants were tested alone and were pseudo-randomly assigned to one of two conditions: depletion or control. They first completed the tasks described in Hagger *et al*.^3^, including the letter ‘e’ task (difficult or easy), four manipulation check questions measuring effort, difficulty, fatigue, and frustration, and the multi-source interference task (MSIT). They then answered the four manipulation check questions again and completed a series of three risk-taking tasks, administered in Qualtrics. In the risky gains task, they chose between receiving a sum of money with certainty and receiving a larger sum with 50% probability. In the risky losses task, they chose between losing a sum of money with certainty and losing a larger sum with 50% probability. In the mixed gambles, they chose to either accept or reject a gamble in which they had a 50/50 chance of receiving/losing a sum of money. Participants were informed that one of their decisions would be randomly selected for actual payment at the end of the experiment.

#### Data analysis

The main analysis of interest investigates the proportion of risky choices in the depletion vs. control condition in the gain domain, loss domain, and mixed gambles, using independent samples t-tests and Mann-Whitney U tests. To confirm the results, we perform regression analyses in which we control for age and gender. We finally investigate the proportion of participants who chose the risky option *on each trial* in the depletion vs. control condition, using chi-square tests. Following Hagger *et al*.^3^, we repeated the analyses while excluding participants (*n* = 3) who had less than 80% correct on the letter ‘e’ task; however, doing so did not change the results and we therefore do not report them in the paper.

### Study 2

The hypotheses, independent and dependent variables, target sample size, and analysis plan were specified in the preregistration^49^. Experimental scripts and data are available at OSF^50^.

#### Participants

Participants were recruited using the same methods as Study 1. A power calculation in G*Power 3.1 indicated that 158 participants were needed to detect an effect size of *d* = 0.4 using an independent samples t-test with 70% power. One participant was excluded due to technical issues administering the Stroop task. The final sample consisted of usable data from 88 males and 88 females (age 24.75 ± 5.79 years [m ± SD]), in addition to 54 participants who did not report age and gender, yielding a total sample of 230 participants. We recruited more participants than we specified in the preregistration, because we expected dropout and because the first 54 participants did not report age and gender due to an error in the script. All participants gave their written informed consent and were compensated with 100 SEK (approx. 12 USD) in addition to the payoff from one randomly selected decision. All methods were carried out in accordance with relevant ethical guidelines and regulations.

#### Materials and procedure

The experiment was conducted in a computer lab in sessions of up to 10 participants. Divider screens prevented participants from seeing each other’s responses. Participants were pseudo-randomly assigned to one of two conditions: ego depletion or control. Participants first completed a Stroop task, adapted from Dang *et al*.^37^ and administered in E-Prime. On each of 256 trials, a color word was presented on the screen. Participants’ task was to indicate the font color of the word by pressing one of four buttons on the keyboard (e.g., “r” if the font color was red). In the depletion condition, 75% of trials were incongruent (i.e., the font color was different from the meaning of the word), while the remaining 25% were congruent (i.e., the font color matched the meaning of the word). In the control condition, all trials were congruent. Following the Stroop task, participants answered the same four manipulation check questions as in Study 1 and completed the risk-taking tasks in Qualtrics. The risk tasks were the same as in Study 1, except we included more trials in the range where there seemed to be a tendency toward an effect in Study 1. Participants were informed that one of their decisions would be randomly selected for actual compensation at the end of the experiment.

#### Data analysis

Data analysis follows the same structure as Study 1. Excluding participants (*n* = 7) with less than 80% correct on the Stroop task did not change the results, so we report only the analyses that include the full sample.

### Study 3

The hypotheses, independent and dependent variables, target sample size, and analysis plan were specified in the preregistration^51^. Experimental scripts and data are available at OSF^50^.

#### Participants

Participants were recruited on Amazon Mechanical Turk (MTurk) for a study entitled “Cognitive Tasks”. A power calculation in G*Power 3.1 indicated that 788 participants were needed in order to detect a small effect size (*d* = 0.2) using a two-tailed independent samples t-test with 80% power. The preregistration specified that we aimed to recruit 1,000 participants in order to account for potential dropout due to technical issues or failure to complete the tasks; however, because the dropout rate initially seemed higher than expected, we increased the sample size to 1,500 in order to ensure sufficient power. Participants were excluded if they did not complete the risk-taking tasks or if they participated more than once (if this were the case, we kept only the first instance). The final sample consisted of usable data from 789 males and 581 females (age 39.77 ± 12.38 years [m ± SD]), in addition to 17 participants who did not report age and gender, yielding a total sample of 1,389 participants. All participants provided informed consent and were compensated with 3 USD in addition to the payoff from one randomly selected decision. All methods were carried out in accordance with relevant ethical guidelines and regulations.

#### Materials and procedure

The experiment was administered in Inquisit 5 (web-based version). Participants were randomly assigned to one of two conditions: ego depletion or control. Participants first completed the letter ‘e’ task, adapted from the Vohs *et al*. replication project^38^. In the first phase, participants were presented with a passage of text and were instructed to delete all instances of the letter ‘e’. Participants worked on this task until they were done or until 7 minutes had passed, whichever came first. In the second phase, participants were presented with a different passage of text. Participants in the control condition were again instructed to delete all instances of the letter ‘e’. Participants in the depletion condition were instructed to delete all instances of the letter ‘e’, except if an ‘e’ was followed by a vowel or if a vowel came two letters before the ‘e’ (not counting spaces). Participants worked on this task until they were done or until 8 minutes had passed, whichever came first. Following the letter ‘e’ task, participants answered the same four manipulation check questions as in Study 1 and 2. They then completed the same risk-taking tasks as in Study 2, except incentives ranged from −1 to 1 USD. Participants were informed that one of their decisions would be randomly selected for actual payment at the end of the experiment.

#### Data analysis

Data analysis follows the same structure as Study 1 and 2. The primary analysis includes all participants who completed the risk tasks, excluding only those who participated more than once (an intent-to-treat analysis; included *n* = 1,389). The secondary analysis additionally excludes participants who did not comply with the instructions in the letter ‘e’ task. Specifically, participants were excluded if they met one or more of the following criteria: (1) they deleted less than 60 (out of 337) e’s or deleted all text in the first phase of the letter ‘e’ task; (2) they deleted less than 60 e’s in the second phase of the letter ‘e’ task (control condition) or they deleted less than 50 e’s and/or deleted more than 11 e’s that were followed by a vowel and therefore should not have been deleted (depletion condition). For participants in the depletion condition who deleted 9–10 e’s followed by a vowel, we manually inspected their response. Excluding participants based on these criteria resulted in a sample size of *n* = 815. Because results remained the same across the primary and secondary analyses, we report only the primary analysis in the paper. Results from the secondary analysis are provided in the Supplemental Materials (see Supplementary Tables S12–S15 and Supplementary Figs S1 and S2).

## Supplementary information

Supplementary Materials

## Data Availability

Data are available at the Open Science Framework (OSF)^50^.

## References

[1] Strömbäck C, Lind T, Skagerlund K, Västfjäll D, Tinghög G (2017). Does self-control predict financial behavior and financial well-being?. J. Behav. Exp. Financ..

[2] Duckworth, A. L., Taxer, J. L., Eskreis-winkler, L., Galla, B. M. & Gross, J. J. Self-control and academic achievement. *Annu Rev Psychol.* **70**, 373–399 (2019). 10.1146/annurev-psych-010418-103230 30609915

[3] Hagger MS (2016). A multilab preregistered replication of the ego-depletion effect. Perspect. Psychol. Sci..

[4] Evans JSBT (2003). In two minds: dual-process accounts of reasoning. Trends Cogn. Sci..

[5] Stanovich, K. E. & West, R. F. Individual differences in reasoning: Implications for the rationality debate? *Behav. Brain Sci*. **23**, 645–726 (2000). 10.1017/s0140525x00003435 11301544

[6] Epstein S (1994). Integration of the cognitive and the psychodynamic unconscious. Am. Psychol..

[7] Kahneman D (2003). Maps of bounded rationality: Psychology for behavioral economics. Am. Econ. Rev..

[8] Kahneman, D. *Thinking, Fast and Slow*. (Farrar, Straus and Giroux, 2011).

[9] Kahneman D, Tversky A (1979). Prospect theory: An analysis of decision under risk. Econometrica.

[10] Kirchler M (2017). The effect of fast and slow decisions on risk taking. J. Risk Uncertain..

[11] Porcelli AJ, Delgado MR (2009). Acute stress modulates risk taking in financial decision making. Psychol. Sci..

[12] Frederick S (2005). Cognitive reflection and decision making. J. Econ. Perspect..

[13] Persson, E., Asutay, E., Hagman, W., Västfjäll, D. & Tinghög, G. Affective response predicts risky choice for fast, but not slow, decisions. *J. Neurosci. Psychol. Econ*. **11**, 213–227 (2018).

[14] Baumeister RF, Bratslavsky E, Muraven M, Tice DM (1998). Ego depletion: Is the active self a limited resource?. J. Pers. Soc. Psychol..

[15] Hagger MS, Wood C, Stiff C, Chatzisarantis NLD (2010). Ego depletion and the strength model of self-control: A meta-analysis. Psychol. Bull..

[16] Dang J, Liu Y, Liu X, Mao L (2017). The ego could be depleted, providing initial exertion is depleting: A preregistered experiment of the ego depletion effect. Soc. Psychol. (Gott)..

[17] Dang J (2018). An updated meta-analysis of the ego depletion effect. Psychol. Res..

[18] Bruyneel SD, Dewitte S, Franses PH, Dekimpe MG (2009). I felt low and my purse feels light: Depleting mood regulation attempts affect risk decision making. J. Behav. Decis. Mak..

[19] Fischer P, Kastenmüller A, Asal K (2012). Ego depletion increases risk-taking. J. Soc. Psychol..

[20] Freeman N, Muraven M (2010). Self-control depletion leads to increased risk taking. Soc. Psychol. Personal. Sci..

[21] Friehe T, Schildberg-Hörisch H (2017). Self-control and crime revisited: Disentangling the effect of self-control on risk taking and antisocial behavior. Int. Rev. Law Econ..

[22] Molet M (2012). Decision making by humans in a behavioral task: Do humans, like pigeons, show suboptimal choice?. Learn. Behav..

[23] Schmeichel BJ, Harmon-Jones C, Harmon-Jones E (2010). Exercising self-control increases approach motivation. J. Pers. Soc. Psychol..

[24] Unger A, Stahlberg D (2011). Ego-depletion and risk behavior: Too exhausted to take a risk. Soc. Psychol. (Gott)..

[25] Yan X-H (2014). The effect of self-control resource on risk preference. Soc. Behav. Personal. an Int. J..

[26] Kostek J, Ashrafioun L (2014). Tired winners: The effects of cognitive resources and prior winning on risky decision making. J. Gambl. Stud..

[27] Imhoff R, Schmidt AF, Gerstenberg F (2014). Exploring the interplay of trait self-control and ego depletion: Empirical evidence for ironic effects. Eur. J. Pers..

[28] Gerhardt H, Schildberg-Hörisch H, Willrodt J (2017). Does self-control depletion affect risk attitudes?. Eur. Econ. Rev..

[29] Giacomantonio M, Jordan J, Fennis BM, Panno A (2014). When the motivational consequences of ego depletion collide: Conservation dominates over reward-seeking. J. Exp. Soc. Psychol..

[30] De Langhe, B., Sweldens, S., van Osselaer, S. M. J. & Turk, M. The emotional information processing system is risk averse: Ego-depletion and investment behavior. *ERIM Rep. Ser*. 1–25 (2008).

[31] Carter EC, McCullough ME (2013). Is ego depletion too incredible Evidence for the overestimation of the depletion effect. Behav. Brain Sci..

[32] Carter EC, McCullough ME (2014). Publication bias and the limited strength model of self-control: has the evidence for ego depletion been overestimated? *Front*. Psychol..

[33] Carter EC, Kofler LM, Forster DE, McCullough ME (2015). A series of meta-analytic tests of the depletion effect: Self-control does not seem to rely on a limited resource. J. Exp. Psychol. Gen..

[34] Baumeister RF, Vohs KD (2016). Misguided effort with elusive implications. Perspect. Psychol. Sci..

[35] Friese, M., Loschelder, D. D., Gieseler, K., Frankenbach, J. & Inzlicht, M. Is ego depletion real? An analysis of arguments, *Pers. Soc. Psychol. Rev.*, **23**, 107–131 (2018). 10.1177/1088868318762183 29591537

[36] Dang J (2016). Commentary: A multilab preregistered replication of the ego-depletion effect. Front. Psychol..

[37] Dang, J. *et al*. Multi-lab replication reveals a small but significant ego depletion effect, 10.31234/osf.io/cjgru (2019).

[38] Vohs, K. D., Schmeichel, B. J. & Baumeister, R. F. A pre-registered depletion replication project: The paradigmatic replication approach. In *Society for Social and Personality Psychology* (2018).

[39] Vadillo MA, Gold N, Osman M (2018). Searching for the bottom of the ego well: failure to uncover ego depletion in Many Labs 3. R. Soc. Open Sci..

[40] Muraven M, Slessareva E (2003). Mechanisms of self-control failure: Motivation and limited resources. Personal. Soc. Psychol. Bull..

[41] Vohs KD, Baumeister RF, Schmeichel BJ (2012). Motivation, personal beliefs, and limited resources all contribute to self-control. J. Exp. Soc. Psychol..

[42] Koppel Lina, Andersson David, Morrison India, Posadzy Kinga, Västfjäll Daniel, Tinghög Gustav (2017). The effect of acute pain on risky and intertemporal choice. Experimental Economics.

[43] Gergelyfi M, Jacob B, Olivier E, Zénon A (2015). Dissociation between mental fatigue and motivational state during prolonged mental activity. Front. Behav. Neurosci..

[44] Vohs KD (2008). Making choices impairs subsequent self-control: A limited-resource account of decision making, self-regulation, and active initiative. J. Pers. Soc. Psychol..

[45] Danziger S, Levav J, Avnaim-Pesso L (2011). Extraneous factors in judicial decisions. Proc. Natl. Acad. Sci..

[46] Persson, E., Barrafrem, K., Meunier, A. & Tinghög, G. The effect of decision fatigue on surgeons’ clinical decision making. (under review). 10.1002/hec.3933 PMC6851887 31344303

[47] Tinghög, G., Koppel, L., Hagger, M. S. & Holcombe, A. O. Fork of RRR - SripadaEtAl2014 Tinghög Lab - Tinghög Replication. Available at: https://osf.io/yi5fm (2018).

[48] Greiner B (2015). Subject pool recruitment procedures: organizing experiments with ORSEE. J. Econ. Sci. Assoc..

[49] Koppel, L. & Tinghög, G. Ego depletion and risk taking (#10597). Available at: https://aspredicted.org/jx28b.pdf (2018). 10.1038/s41598-019-46103-0 PMC6611900 31278364

[50] Koppel, L. & Tinghög, G. Ego depletion and risk taking. Available at: https://osf.io/8pm5k (2018). 10.1038/s41598-019-46103-0 PMC6611900 31278364

[51] Koppel, L. & Tinghög, G. Ego depletion and risk taking - mturk study (#22788). Available at: https://aspredicted.org/rk9um.pdf (2018).

