# Supplementary material for: No Effect of Ego Depletion on Risk Taking
Source: Sci Rep. 2019 Jul 5;9:9724. doi: 10.1038/s41598-019-46103-0 (PMC6611900; doi:10.1038/s41598-019-46103-0)
Supplement: Supplementary file 1 — Supplementary Materials [file 41598_2019_46103_MOESM1_ESM.pdf]

Supplemental Materials

No Effect of Ego Depletion on Risk Taking

Lina Koppel[1,2], David Andersson[1,2], Daniel Västfjäll[1,2,3,4], Gustav Tinghög[1,2,5]*

[1]JEDI Lab, Division of Economics, Department of Management and Engineering, Linköping University, Linköping, Sweden
[2]Center for Social and Affective Neuroscience, Department of Clinical and Experimental Medicine, Linköping University, Linköping, Sweden
[3]Division of Psychology, Department of Behavioral Sciences and Learning, Linköping University, Linköping, Sweden
[4]Decision Research, Eugene, OR, USA
[5]The National Center for Priority Setting in Health Care, Department of Medical and Health Sciences, Linköping University, Linköping, Sweden

***Correspondence:** gustav.tinghog@liu.se

Table S1

Overview of previous studies on ego depletion and risk taking

**Author(s)**	**N**	**Depleting task**	**Dependent measure**	**Incentivized**	**Effect of ego depletion**
Bruyneel et al. (2008; study 2)	71	Stroop	Lottery ticket expenditures	No	Increased risk taking (*p* = .03)
De Langhe et al. (2008)	64	Stroop	Investment game	No	No overall effect, but a 3-way interaction (*p* = .05) indicating that ego depletion increased risk taking in the second half of the task in participants who reported relying more on intuition
	57	Stroop	Iowa Gambling Task (Bechara et al., 1994)	No	No overall effect, but improved performance (i.e., reduced risk taking) in the first half of the task (*p* = .05)
Fischer et al. (2012)	33	Thought suppression	Sensation-seeking questionnaire (Zukerman, 1971)	No	Increased sensation seeking (*p* = .007)
	30	Emotion suppression	Vienna Risk Taking Test (Schuhfried, 2006)	No	Increased risk taking (*p* = .04)
	74	Though suppression; emotion suppression	Domain-Specific Risk-Taking Scale (DOSPERT; Weber et al., 2002)	No	(Marginally) increased (self-reported) risk-taking (*p* = .09)
	38	Emotion suppression	DOSPERT (Weber et al., 2002)	No	Increased (self-reported) risk taking (*p* = .04)
Freeman & Muraven (2010)	70	Crossing out letters	Choice Dilemma Questionnaire (Kogan & Wallach, 1964)	No	Increased risk taking (*p* < .05)
	46	Attention video	Balloon Analog Risk Task (BART; Lejuez et al., 2002)	Yes	Increased risk taking (*p* < .05)
Friehe & Schildberg-Hörisch (2017)	90	Crossing out letters	Investment task	Yes	(Marginally) increased risk taking (*p* < .10)
Gerhardt et al. (2017)	308	Crossing out letters	Choice lists	Yes	No effect (*p*s > .05)
Giacomantonio et al. (2014)	92	Attention essay	BART (Lejuez et al., 2002)	Yes	(Marginally) increased risk taking in the regular BART (*p* = .055) and (marginally) reduced risk taking in a version of BART that requires physical effort (*p* = .052)
Imhoff et al. (2014; study 2)	127	Working memory task	Game of dice task	No	Increased risk taking (*p* < .01)
Kostek & Ashrafloun (2013)	81	Attention essay	Betting task	Yes	Reduced risk taking (*p* < .05)

Molet et al. (2012; study 2)	30	Attention video	Space-war videogame	No	Increased risk taking (*p* = .049)
Schmeichel et al. (2010; study 2b)	132	Attention essay	Coin tossing task	No	Increased risk taking (*p* = .02)
Unger & Stahlberg (2011)	150	Vocabulary-learning	Investment scenario	Yes	Reduced risk taking (*p* = .04)
	48	Vocabulary-learning	Investment scenario	Yes	Reduced risk taking (*p* = .04)
	81	Emotion suppression	Investment scenario	Yes	Reduced risk taking (*p* = .01)
Yan (2014; study 2)	55	Crossing out letters	Binary choice between safe and risky option	No	Reduced risk taking (*p* = .035)

Table S2

Means and 95% confidence intervals (CIs) for the manipulation check questions in Study 1

	Depletion (n = 48)		Control (n = 49)		Independent samples t-test		Mann-Whitney U	
	M	[95% CI]	M	[95% CI]	*t*	*p*	Z	*p*
First assessment								
Effort	5.85	[5.59, 6.12]	4.84	[4.34, 5.33]	3.63	<.001	2.85	.004
Difficulty	4.40	[4.02, 4.77]	1.96	[1.74, 2.18]	11.28	<.001	7.67	<.001
Fatigue	3.42	[3.00, 3.83]	3.04	[2.61, 3.47]	1.26	.209	1.15	.250
Frustration	3.27	[2.78, 3.76]	2.14	[1.77, 2.52]	3.67	<.001	3.53	<.001
Second assessment								
Effort	5.63	[5.22, 6.03]	5.47	[5.07, 5.87]	0.55	.583	–0.62	.535
Difficulty	3.75	[3.21, 4.29]	4.55	[4.18, 4.92]	–2.49	.015	–2.21	.027
Fatigue	4.48	[4.03, 4.93]	4.37	[3.90, 4.84]	0.345	.731	–0.29	.774
Frustration	3.38	[2.79, 3.96]	3.78	[3.35, 4.20]	–1.12	.267	–1.40	.161

Table S3

Regression analyses of risky choices in Study 1

	Gain		Loss		Mixed	
	(1)	(2)	(1)	(2)	(1)	(2)
Depletion	0.042<br>(0.046)	0.031<br>(0.057)	0.014<br>(0.041)	0.004<br>0.043)	0.049<br>(0.042)	0.033<br>(0.050)
Female	0.104*<br>(0.048)	0.088<br>(0.069)	0.079<br>(0.049)	0.064<br>(0.070)	–0.059<br>(0.046)	–0.082<br>(0.065)
Depletion × Female		0.034<br>(0.096)		0.032<br>(0.097)		0.049<br>(0.093)
Age	0.005<br>(0.010)	0.005<br>(0.010)	0.004<br>(0.009)	0.003<br>(0.009)	–0.000<br>(0.009)	–0.001<br>(0.009)
Constant	0.495*<br>(0.224)	0.506*<br>(0.229)	0.225<br>(0.210)	0.234<br>(0.210)	0.493*<br>(0.207)	0.508*<br>(0.206)

Notes. This table reports OLS coefficient estimates (robust standard errors in parentheses). The dependent variable is the proportion of risky choices. “Depletion” is a dummy for the depletion condition. “Female” is a gender dummy. “Pain × Female” allows the effect of pain to differ between the two genders. “Age” is the participant’s age in years.

* $p < .05$, ** $p < .01$, *** $p < .001$

Table S4

Frequency (percent) of risky choices per trial in the gain domain, loss domain, and mixed gambles, as a function of condition (depletion vs. control) in Study 1

	Depletion (n = 48)	Control (n = 49)	Chi-square test *p*
Gain domain			
20 vs. 100/0	45 (93.8%)	47 (95.9%)	.629[a]
30 vs. 100/0	44 (91.7%)	46 (93.9%)	.674[a]
40 vs. 100/0	44 (91.7%)	42 (85.7%)	.355[a]
50 vs. 100/0	21 (43.8%)	17 (34.7%)	.361
60 vs. 100/0	11 (22.9%)	7 (14.3%)	.274
Loss domain			
–20 vs. -100/0	3 (6.3%)	3 (6.1%)	.979[a]
–30 vs. –100/0	3 (6.3%)	3 (6.1%)	.979[a]
–40 vs. –100/0	5 (10.4%)	7 (14.3%)	.563
–50 vs. –100/0	28 (58.3%)	23 (46.9%)	.261
–60 vs. –100/0	44 (91.7%)	46 (93.9%)	.674[a]
Mixed gambles			
0 vs. 80/–20	47 (97.9%)	47 (95.9%)	.570[a]
0 vs. 80/–40	40 (83.3%)	39 (79.6%)	.636
0 vs. 80/–60	26 (54.2%)	22 (44.9%)	.361
0 vs. 80/–80	8 (16.7%)	6 (12.2%)	.536
0 vs. 80/–100	3 (6.3%)	0 (0.0%)	.076[a]

[a] Fisher's exact test

Table S5

Means and 95% confidence intervals (CIs) for the manipulation check questions in Study 2

	Depletion (n = 114)		Control (n = 116)		Independent samples t-test		Mann-Whitney U	
	M	[95% CI]	M	[95% CI]	*t*	*p*	Z	*p*
Effort	5.22	[4.99, 5.45]	4.41	[4.12, 4.71]	4.24	<.001	3.82	<.001
Difficulty	4.07	[3.82, 4.32]	2.67	[2.40, 2.94]	7.45	<.001	7.05	<.001
Fatigue	3.88	[3.59, 4.16]	3.47	[3.18, 3.75]	2.02	.045	2.17	.030
Frustration	3.26	[2.96, 3.56]	3.39	[3.06, 3.72]	–0.56	.579	–0.40	.687

Table S6

Regression analyses of risky choices in Study 2

	Gain		Loss		Mixed	
	(1)	(2)	(1)	(2)	(1)	(2)
Depletion	–0.010<br>(0.043)	–0.008<br>(0.056)	–0.049<br>(0.039)	–0.049<br>(0.051)	–0.003<br>(0.035)	0.018<br>(0.048)
Female	0.060<br>(0.043)	0.062<br>(0.061)	–0.029<br>(0.038)	–0.029<br>(0.054)	–0.093**<br>(0.034)	–0.072<br>(0.047)
Depletion × Female		–0.004<br>(0.087)		0.000<br>(0.077)		–0.042<br>(0.069)
Age	0.000<br>(0.004)	0.000<br>(0.004)	0.002<br>(0.039)	0.002<br>(0.054)	–0.001<br>(0.003)	–0.001<br>(0003)
Constant	0.521***<br>(0.104)	0.520***<br>(0.103)	0.441***<br>(0.097)	0.441***<br>(0.098)	0.532***<br>(0.073)	0.524***<br>(0.072)

Notes. This table reports OLS coefficient estimates (robust standard errors in parentheses). The dependent variable is the proportion of risky choices. “Depletion” is a dummy for the depletion condition. “Female” is a gender dummy. “Pain × Female” allows the effect of pain to differ between the two genders. “Age” is the participant’s age in years.

* $p < .05$, ** $p < .01$, *** $p < .001$

Table S7

Frequency (percent) of risky choices per trial in the gain domain, loss domain, and mixed gambles, as a function of condition (depletion vs. control) in Study 2

	Depletion (n = 114)	Control (n = 116)	Chi-square test *p*
Gain domain			
35 vs. 100/0	104 (91.2%)	108 (93.1%)	.597
40 vs. 100/0	96 (84.2%)	101 (87.1%)	.536
45 vs. 100/0	85 (74.6%)	89 (76.7%)	.702
50 vs. 100/0	46 (40.4%)	51 (44.0%)	.579
55 vs. 100/0	35 (30.7%)	41 (35.3%)	.454
60 vs. 100/0	24 (21.1%)	22 (19.0%)	.692
45 vs. 90/0	57 (50.0%)	60 (51.7%)	.794
Loss domain			
–35 vs. –100/0	10 (8.8%)	11 (9.5%)	.852
–40 vs. –100/0	18 (15.8%)	21 (18.1%)	.640
–45 vs. –100/0	28 (24.6%)	37 (31.9%)	.217
–50 vs. –100/0	53 (46.5%)	65 (56.0%)	.148
–55 vs. –100/0	82 (71.9%)	93 (80.2%)	.143
–60 vs. –100/0	98 (86.0%)	103 (88.8%)	.518
–45 vs. –90/0	42 (36.8%)	54 (46.6%)	.135
Mixed gambles			
0 vs. 80/–20	110 (96.5%)	114 (98.3%)	.396
0 vs. 80/–40	90 (78.9%)	96 (82.8%)	.463
0 vs. 80/–50	58 (50.9%)	68 (58.6%)	.238
0 vs. 80/–60	47 (41.2%)	46 (39.7%)	.808
0 vs. 80/–70	32 (28.1%)	28 (24.1%)	.497
0 vs. 80/–80	23 (20.2%)	19 (16.4%)	.456
0 vs. 80/–100	8 (7.0%)	6 (5.2%)	.558

Table S8

Regression analyses of risky choices in the pooled data from Study 1 and 2

	Gain	Loss	Mixed
Depletion	0.622	–0.027	0.015
	(.032)	(.029)	(.027)
Female	0.008*	0.007	–0.082**
	(.033)	(.031)	(.028)
Age	0.075	0.002	–0.001
	(.004)	(.004)	(.003)
Study	0.001***	0.097**	–0.015
	(.032)	(.029)	(.028)
Constant	–0.128***	0.311***	0.533***
	(.091)	(.088)	(.069)

Notes. This table reports OLS coefficient estimates (robust standard errors in parentheses). The dependent variable is the proportion of risky choices. “Depletion” is a dummy for the depletion condition. “Female” is a gender dummy. “Age” is the participant’s age in years. “Study” is a dummy for Study 2.

* $p < .05$, ** $p < .01$, *** $p < .001$

Table S9

Means and 95% confidence intervals (CIs) for the manipulation check questions in Study 3 (primary analysis; n = 1,389)

	Depletion (n = 673)		Control (n = 716)		Independent samples t-test		Mann-Whitney U	
	M	[95% CI]	M	[95% CI]	*t*	*p*	Z	*p*
Effort	6.08	[5.98, 6.19]	5.98	[5.87, 6.10]	1.24	.215	0.44	.661
Difficulty	4.85	[4.72, 4.98]	4.01	[3.85, 4.16]	8.11	<.001	7.45	<.001
Fatigue	4.75	[4.61, 4.90]	4.53	[4.39, 4.68]	2.09	.037	2.05	.040
Frustration	4.88	[4.73, 5.02]	4.52	[4.37, 4.68]	3.27	.001	2.91	.004

Table S10

Regression analyses of risky choices in Study 3 (primary analysis; n = 1,370)

	Gain		Loss		Mixed	
	(1)	(2)	(1)	(2)	(1)	(2)
Depletion	-0.016	-0.007	-0.027	-0.042	0.005	0.018
	(0.019)	(0.026)	(0.019)	(0.026)	(0.018)	(0.024)
Female	-0.040*	-0.030	0.034	0.017	0.017	0.032
	(0.020)	(0.027)	(0.019)	(0.027)	(0.018)	(0.025)
Depletion × Female		-0.021		0.036		-0.031
		(0.039)		(0.039)		(0.036)
Age	-0.001	-0.001	0.001	0.001	-0.001	-0.001
	(0.001)	(0.001)	(0.001)	(0.001)	(0.001)	(0.001)
Constant	0.609***	0.605***	0.441***	0.448***	0.532***	0.526***
	(0.035)	(0.037)	(0.036)	(0.037)	(0.033)	(0.034)

Notes. This table reports OLS coefficient estimates (robust standard errors in parentheses). The dependent variable is the proportion of risky choices. “Depletion” is a dummy for the depletion condition. “Female” is a gender dummy. “Pain × Female” allows the effect of pain to differ between the two genders. “Age” is the participant’s age in years.

* $p < .05$, ** $p < .01$, *** $p < .001$

Table S11

Frequency (percent) of risky choices per trial in the gain domain, loss domain, and mixed gambles, as a function of condition (depletion vs. control) in Study 3 (primary analysis; n = 1,389)

	Depletion (n = 673)	Control (n = 716)	Chi-square test *p*
Gain domain			
35 vs. 100/0	544 (80.8%)	587 (82.0%)	.581
40 vs. 100/0	461 (68.5%)	497 (69.4%)	.713
45 vs. 100/0	425 (63.2%)	464 (64.8%)	.521
50 vs. 100/0	297 (44.1%)	317 (44.3%)	.957
55 vs. 100/0	246 (36.6%)	272 (38.0%)	.580
60 vs. 100/0	199 (29.6%)	210 (29.3%)	.922
45 vs. 90/0	349 (51.9%)	392 (54.8%)	.280
Loss domain			
–35 vs. –100/0	185 (27.5%)	223 (31.2%)	.135
–40 vs. –100/0	233 (34.6%)	261 (36.5%)	.476
–45 vs. –100/0	260 (38.6%)	316 (44.1%)	.038
–50 vs. –100/0	374 (55.6%)	415 (58.0%)	.369
–55 vs. –100/0	437 (64.9%)	487 (68.0%)	.224
–60 vs. –100/0	478 (71.0%)	534 (74.6%)	.136
–45 vs. –90/0	298 (44.3%)	334 (46.7%)	.376
Mixed gambles			
0 vs. 80/–20	572 (85.0%)	614 (85.8%)	.688
0 vs. 80/–40	479 (71.2%)	508 (71.0%)	.927
0 vs. 80/–50	381 (56.6%)	400 (55.9%)	.779
0 vs. 80/–60	290 (43.1%)	307 (42.9%)	.936
0 vs. 80/–70	255 (37.9%)	266 (37.2%)	.776
0 vs. 80/–80	240 (35.7%)	262 (26.6%)	.718
0 vs. 80/–100	185 (27.5%)	194 (27.1%)	.869

Table S12

Means and 95% confidence intervals (CIs) for the manipulation check questions in Study 3 (seconday analysis; n = 815)

	Depletion (n = 352)		Control (n = 463)		Independent samples t-test		Mann-Whitney U	
	M	[95% CI]	M	[95% CI]	*t*	*p*	Z	*p*
Effort	6.45	[6.36, 6.55]	6.46	[6.38, 6.55]	-0.15	0.878	-0.81	0.419
Difficulty	4.72	[4.55, 4.89]	3.85	[3.66, 4.04]	6.76	<.001	6.04	<.001
Fatigue	4.67	[4.48. 4.86]	4.55	[4.38, 4.72]	0.93	0.354	1.07	0.284
Frustration	4.70	[4.51, 4.90]	4.40	[4.21, 4.58]	2.23	0.026	1.95	0.052

Table S13

Significance tests of the difference in the proportion of risky choices in the depletion vs. control condition in the gain domain, loss domain, and mixed gambles in Study 3 (secondary analysis; n = 815)

	Depletion		Control		Independent-samples t-test			Mann-Whitney U
	M	[95% CI]	*M*	[95% CI]	*t*	*p*	*d*	*p*
Gain	0.53	[0.34, 0.39]	0.55	[0.34, 0.39]	-0.74	.458	0.05	.469
Loss	0.47	[0.33, 0.38]	0.51	[0.33, 0.38]	-1.57	.117	0.11	.124
Mixed	0.47	[0.30, 0.35]	0.46	[0.30, 0.34]	0.11	.914	0.007	.927

Table S14

Regression analyses of risky choices in Study 3 (secondary analysis; n = 810)

	Gain		Loss		Mixed	
	(1)	(2)	(1)	(2)	(1)	(2)
Depletion	-0.020	-0.021	-0.031	-0.040	0.009	0.017
	(0.026)	(0.033)	(0.025)	(0.032)	(0.023)	(0.030)
Female	-0.004	-0.005	0.052*	0.042	0.025	0.035
	(0.026)	(0.034)	(0.025)	(0.033)	(0.023)	(0.030)
Depletion × Female		0.004		0.023		-0.022
		(0.053)		(0.052)		(0.047)
Age	-0.001	-0.001	0.002*	0.002*	0.0002	0.0002
	(0.001)	(0.001)	(0.001)	(0.001)	(0.001)	(0.001)
Constant	0.545***	0.586***	0.404***	0.408***	0.445***	0.441***
	(0.047)	(0.048)	(0.046)	(0.047)	(0.043)	(0.044)

Notes. This table reports OLS coefficient estimates (robust standard errors in parentheses). The dependent variable is the proportion of risky choices. “Depletion” is a dummy for the depletion condition. “Female” is a gender dummy. “Pain × Female” allows the effect of pain to differ between the two genders. “Age” is the participant’s age in years.

* $p < .05$, ** $p < .01$, *** $p < .001$

Table S15

Frequency (percent) of risky choices per trial in the gain domain, loss domain, and mixed gambles, as a function of condition (depletion vs. control) in Study 3 (secondary analysis; n = 815)

	Depletion (n = 352)	Control (n = 463)	Chi-square test *p*
Gain domain			
35 vs. 100/0	292 (83.0%)	388 (83.8%)	.747
40 vs. 100/0	247 (70.2%)	325 (70.2%)	.994
45 vs. 100/0	225 (63.9%)	306 (66.1%)	.520
50 vs. 100/0	149 (42.3%)	203 (43.8%)	.665
55 vs. 100/0	119 (33.8%)	177 (38.2%)	.194
60 vs. 100/0	95 (27.0%)	126 (27.2%)	.943
45 vs. 90/0	178 (50.6%)	253 (54.6%)	.248
Loss domain			
–35 vs. –100/0	74 (21.0%)	129 (27.9%)	.025
–40 vs. –100/0	106 (30.1%)	161 (34.8%)	.160
–45 vs. –100/0	133 (37.8%)	200 (43.2%)	.120
–50 vs. –100/0	190 (54.0%)	270 (58.3%)	.216
–55 vs. –100/0	238 (67.6%)	319 (68.9%)	.696
–60 vs. –100/0	261 (74.2%)	360 (77.8%)	.231
–45 vs. –90/0	157 (44.6%)	213 (46.0%)	.691
Mixed gambles			
0 vs. 80/–20	309 (87.8%)	409 (88.3%)	.809
0 vs. 80/–40	243 (69.0%)	318 (68.7%)	.915
0 vs. 80/–50	190 (54.0%)	242 (52.3%)	.628
0 vs. 80/–60	132 (37.5%)	170 (36.7%)	.819
0 vs. 80/–70	109 (31.0%)	139 (30.0%)	.772
0 vs. 80/–80	98 (27.8%)	136 (29.4%)	.632
0 vs. 80/–100	70 (20.0%)	92 (20.0%)	.996

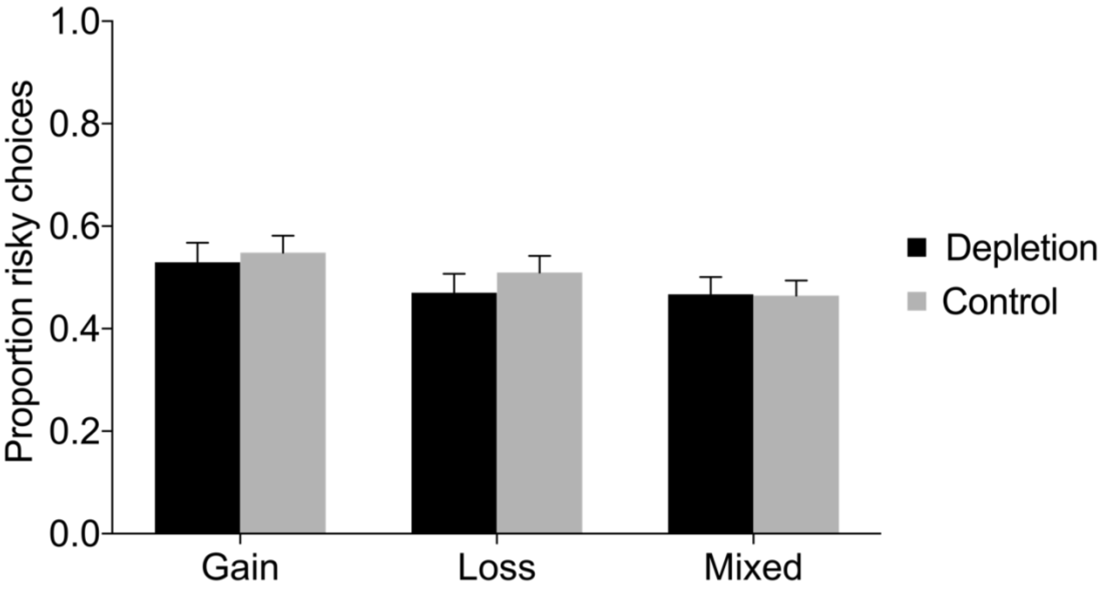

Figure S1. Proportion of risky choices in the gain domain, loss domain, and mixed gambles, as a function of condition (depletion vs. control) in Study 3 (secondary analysis; n = 815). Error bars represent 95% CIs.

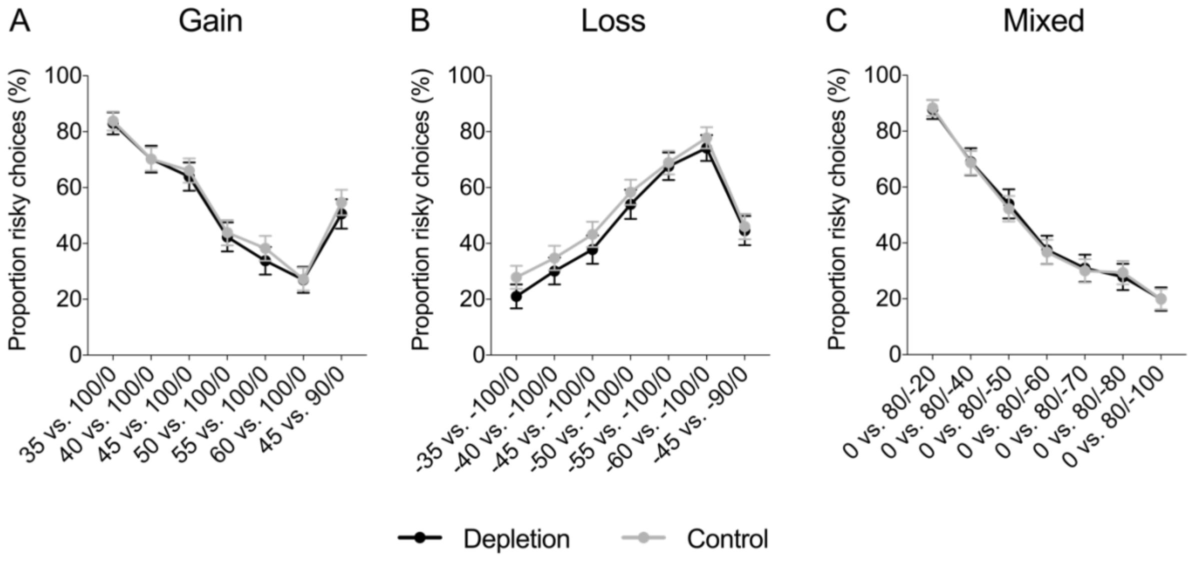

Figure S2. Proportion (%) of risky choices per trial in the (a) gain domain, (b) loss domain, and (c) mixed gambles, as a function of condition (depletion vs. control) in Study 3 (secondary analysis; *n* = 815). Error bars represent 95% CIs.
